# Medial Patellofemoral Ligament Reconstruction Using Gracilis With Pes Anserinus Preservation and Adductor Magnus Pulley

**DOI:** 10.1002/atn2.70214

**Published:** 2026-07-31

**Authors:** Alexandre Coelho, Sergi Gil‐González, Giovanni Grillo, Patrícia Martínez‐Grau, Xavier Pelfort

**Affiliations:** ^1^ Department of Orthopedic Surgery and Traumatology Parc Taulí Hospital Universitari Institut d’Investigació I Innovació Parc Tauli (I3PT‐CERCA) Universitat Autònoma de Barcelona Sabadell Spain

## Abstract

Recurrent patellar instability is a common orthopaedic condition, particularly in young and active patients. Medial patellofemoral ligament (MPFL) reconstruction has emerged as the gold standard for surgical management in cases of objective instability. In this technical note, a quasianatomic MPFL reconstruction is described using an autologous gracilis tendon with preservation of its distal insertion. The graft is passed behind the adductor magnus to replicate the native femoral MPFL path, avoiding femoral tunnel drilling. It is passed in a subretinacular plane and fixed to the patella through convergent bone tunnels using high‐strength sutures (FiberWire; Arthrex, Naples, FL). This minimally invasive, implant‐free technique preserves graft vascularity, reduces surgical morbidity, and offers a biologically favorable alternative to traditional MPFL reconstruction, particularly in skeletally immature patients.

## Graphical abstract

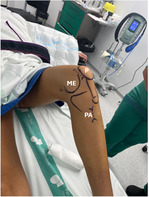

**VIDEO 1:** Surgical technique for medial patellofemoral ligament reconstruction using a gracilis autograft with preservation of the pes anserinus insertion and use of the adductor magnus tendon as a pulley for femoral fixation. Video content can be viewed at https://doi.org/10.1002/atn2.70214.

Patellofemoral instability is a common and disabling condition, especially in young and active individuals, often leading to pain, reduced activity, and early osteoarthritis.^1^ The medial patellofemoral ligament (MPFL) is the primary passive stabilizer of the patella in early flexion, and its disruption—most commonly at the femoral origin—occurs in over 90% of lateral patellar dislocations.^2^

Accurate anatomic MPFL reconstruction remains challenging due to individual variability in femoral attachment, and misplacement of femoral tunnels can compromise graft function.^3^ Traditional techniques often involve complete tendon harvest, femoral drilling, and hardware use, which may increase morbidity and affect graft biology.^3^, ^4^

We describe a quasianatomic MPFL reconstruction using the autologous gracilis tendon (GT) with preservation of its distal insertion. This approach avoids femoral tunnel drilling, preserves graft vascularity, and reduces surgical morbidity. The graft is routed extra‐articularly between layers 2 and 3 and passed behind the adductor magnus to simulate native MPFL biomechanics. This simplified, implant‐free technique offers a biologically favorable and reproducible alternative to conventional methods.

## SURGICAL TECHNIQUE

### Indications

This technique is indicated for patients with objective recurrent lateral patellar instability due to MPFL insufficiency. However, certain anatomical factors—such as patella alta, trochlear dysplasia, coronal malalignment, or an increased tibial tubercle‐trochlear groove  distance—should be carefully assessed, as they may warrant additional procedures in conjunction with MPFL reconstruction to adequately address the underlying instability. The procedure is particularly suited for the following:

• Adolescents or young adults with recurrent dislocations and preserved bony alignment
• Patients with open physes, in whom femoral tunnel drilling or hardware use is undesirable
• Patients with previous soft‐tissue procedures or surgeries, where minimal dissection and tissue preservation are prioritized

A complete illustration of the surgical technique described in this section can be viewed in Video 1.

### Patient Positioning and Setup

**FIGURE 1:**
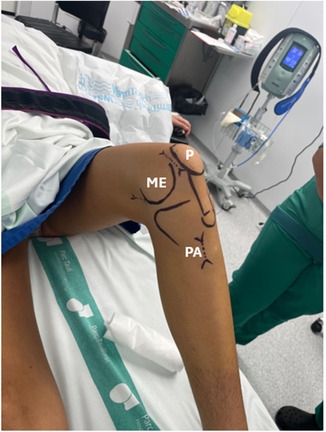
Patient is positioned supine (left knee). Key landmarks for MPFL reconstruction. The ME, P, and PA regions are marked. (ME, medial femoral epicondyle; MPFL, medial patellofemoral ligament; P, patella; PA, pes anserinus.)

The patient is positioned supine with a lateral post and a roll under the knee to allow free flexion and extension. A tourniquet is applied to the upper thigh but is not routinely inflated. Standard sterile draping is performed. Anatomical landmarks (patella, medial epicondyle, and pes anserinus) are marked (Figure 1).

### Graft Harvesting

A 2‐ to 3‐cm oblique skin incision is made over the medial aspect of the knee, centered at the palpable medial epicondyle. After incising the skin and subcutaneous tissue, the deep fascia is carefully opened in line with the incision.

**FIGURE 2:**
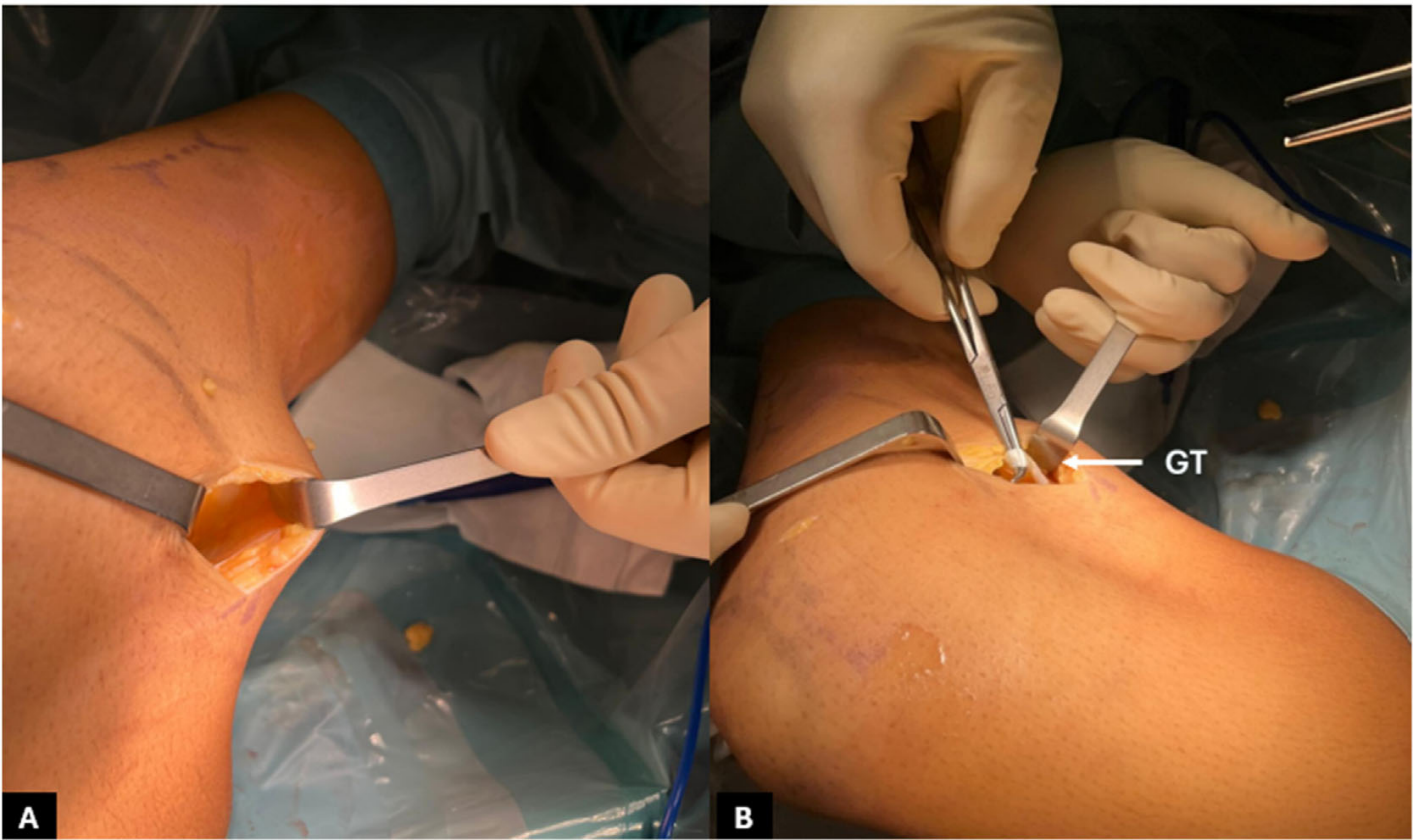
Surgical approach and graft identification, medial view of left knee. (A) Oblique skin incision centered over the medial femoral epicondyle. (B) Intraoperative identification of the GT, recognized as the most anterior and cord‐like of the pes anserinus tendons. (GT, gracilis tendon.)

With the fascia opened, digital palpation is used to identify the tendinous structures of the pes anserinus. The most anterior structure, corresponding to the GT, is palpated as a taut, cord‐like band. Once identified, a loop of number 1 Vicryl (Ethicon, Somerville, NJ) is passed around the GT to aid in manipulation and isolation (Figure 2). Care must be taken not to confuse it with the semimembranosus tendon, which lies deeper and is broader and more rigid.

Blunt dissection is performed proximally and distally to free the tendon and release any vincula or adhesions. Care is taken to preserve the tibial insertion of the GT on the pes anserinus. With the tendon mobilized, an open‐ended tendon stripper is introduced proximally, and the graft is carefully harvested from distal to proximal.

**FIGURE 3:**
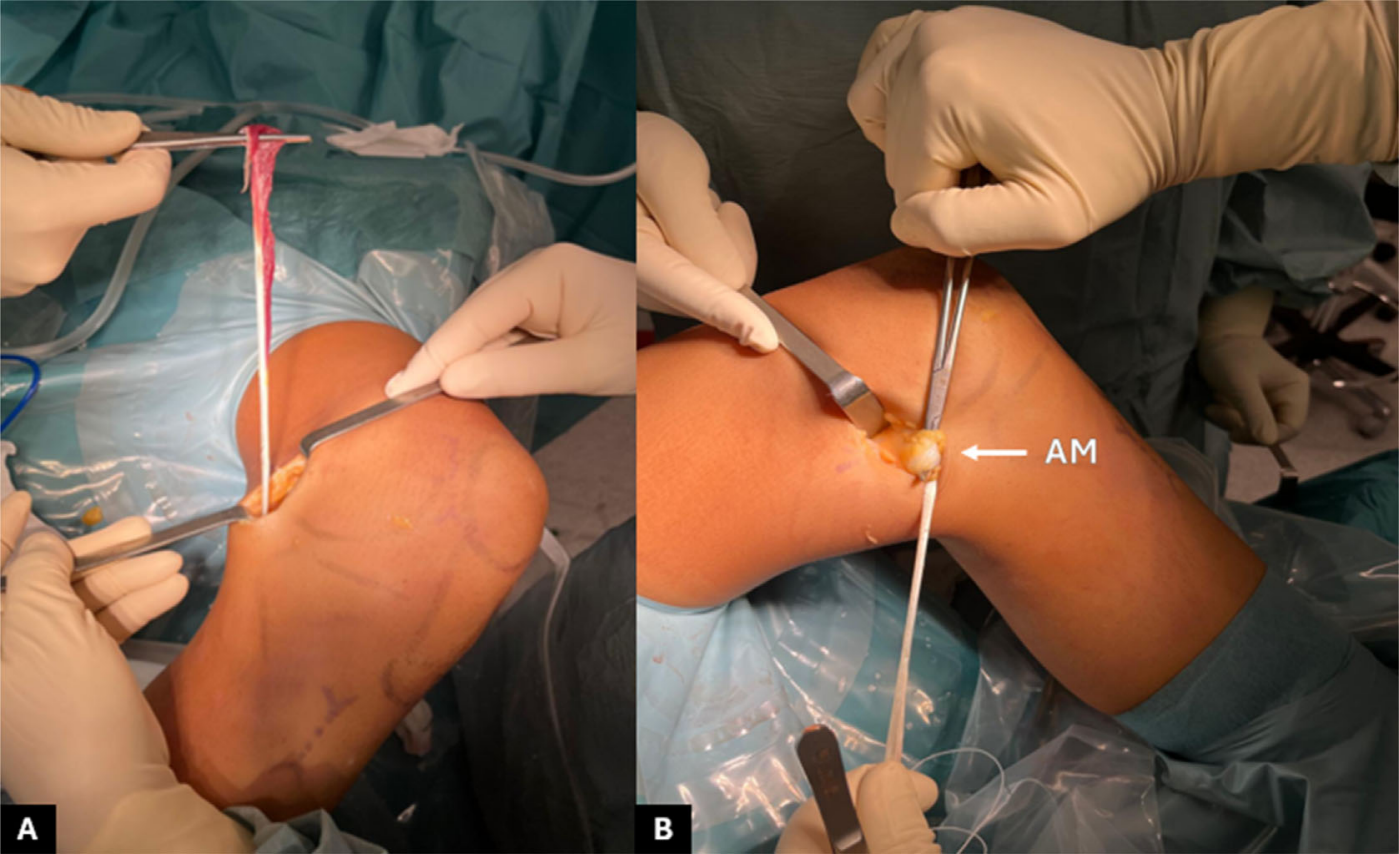
Graft harvesting and passage behind the adductor magnus ‐ left knee. (A) Anterior view. Proximal harvesting of the gracilis tendon using an open‐ended tendon stripper while preserving its tibial insertion. (B) Medial view. Identification of the AM tendon proximal to the adductor tubercle and passage of the graft posterior to it, creating a pulley effect. (AM, adductor magnus.)

The harvested portion is then prepared with the proximal free end of the tendon being tubularized using a high‐strength nonabsorbable suture (FiberWire; Arthrex, Naples, FL) with a Krackow technique, ensuring secure handling and fixation during graft passage (Figure 3).

### Patellar Tunnel Preparation

A 2‐cm vertical incision is made directly over the medial border of the patella, centered on its proximal half. The subcutaneous tissue is bluntly dissected, and the medial retinaculum is opened to expose the medial aspect of the patella.

**FIGURE 4:**
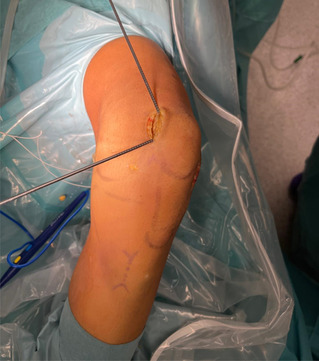
Creation of patellar tunnels, anterior view of left knee. Two converging 4.5 mm bone tunnels are drilled into the patella at the proximal and central thirds, leaving a minimum 10 mm cortical bridge to reduce the risk of fracture.

Two converging 4.5‐mm tunnels are drilled from the medial edge of the patella toward its center, forming a V‐shaped configuration. Care is taken to preserve a cortical bone bridge of at least 10 mm between the tunnels to minimize the risk of iatrogenic patellar fracture (Figure 4).

Once the tunnels are created, a number 1 Vicryl suture (Ethicon, Somerville, NJ) is passed through them to serve as a shuttle for later graft passage. Optionally, a curved dissector may be inserted through the tunnels to facilitate suture or graft passage and ensure smooth transitions.

At this stage, the tunnels are ready to receive the free end of the gracilis graft.

### Adductor Magnus Identification and Graft Passage

Unlike traditional techniques that require femoral tunnel drilling, this procedure uses the adductor magnus tendon as a soft tissue pulley to replicate the femoral attachment of the MPFL.

Through the proximal medial incision (used for gracilis graft harvesting), digital palpation is used to identify the adductor magnus tendon proximal to the adductor tubercle. It is recognized as a thick, cord‐like structure running longitudinally along the posteromedial femur. Once located, the gracilis graft is carefully passed posterior to the adductor magnus tendon, creating a pulley effect that approximates the direction of the native MPFL fibers.

Subsequently, from the medial patellar incision, a curved blunt dissector is introduced to develop a plane between layers 2 and 3 of the medial retinaculum. The dissector is advanced proximally toward the adductor region. Proper positioning is confirmed when the tip of the dissector becomes palpable or visible near the adductor tubercle.

The free end of the gracilis graft, already looped behind the adductor magnus, is then guided anteriorly and retrieved through the plane created between layers 2 and 3, exiting at the medial patellar incision. This subretinacular route reproduces the anatomic course of the native MPFL and maintains the graft in an extra‐articular position, avoiding joint penetration and preserving surrounding structures.

An additional fixation at the adductor magnus is not strictly required, as routing the graft behind the tendon already provides a functional soft tissue pulley that effectively anchors the graft. However, some surgeons may choose to add a supplementary suture at this level to enhance stability and reduce potential micromotion. This step is optional and typically guided by individual surgeon preference. The step‐by‐step description is summarized in Table 1.

**TABLE 1:** Step‐by‐Step Description of the Technique

	**Description**
1	**Incision and exposure**: A 2‐3 cm oblique incision centered on the medial epicondyle is made. The fascia is opened to palpate the medial epicondyle and adductor tubercle
2	**Identification of gracilis tendon**: Palpated as the most anterior, cord‐like structure among the hamstrings
3	**Proximal dissection**: The tendon is dissected proximally and distally while preserving its tibial insertion
4	**Tendon harvest**: The gracilis is harvested using an open tendon stripper. The free end is tubularized with high‐strength sutures (FiberWire; Arthrex, Naples, FL)
5	**Adductor magnus identification**: Through the same incision, the adductor magnus is palpated proximal to the adductor tubercle
6	**Graft passage behind adductor magnus**: The graft is passed posterior to the adductor magnus tendon to simulate a femoral pulley
7	**Subretinacular tunnel creation**: From the patellar incision, a blunt dissector is passed between layers 2 and 3 toward the adductor region to create a graft path
8	**Graft retrieval**: The graft is retrieved distally into the patellar incision via the subretinacular plane
9	**Patellar tunnel preparation**: Two convergent 4.5‐mm tunnels are drilled in the proximal and central thirds of the patella, leaving a ≥10 mm cortical bridge
10	**Graft fixation**: The graft is passed through the patellar tunnels using a Vicryl (Ethicon, Somerville, NJ) shuttle and fixed with high‐strength nonabsorbable sutures (FiberWire; Arthrex, Naples, FL)
11	**Final assessment**: Patellar tracking is evaluated through full range of motion. Overtensioning must be avoided to prevent medial overconstraint and altered patellar tracking

### Fixing the Graft

The previously placed Vicryl (Ethicon, Somerville, NJ) suture—threaded through the 2 convergent patellar tunnels—serves to guide the free proximal end of the gracilis graft through the tunnel system. The graft is passed proximal to distal, from the medial edge of the patella into the converging bony channels. Subsequently, some flexion and extension cycles of the knee are performed to ensure correct graft positioning and prevent protrusion.

**FIGURE 5:**
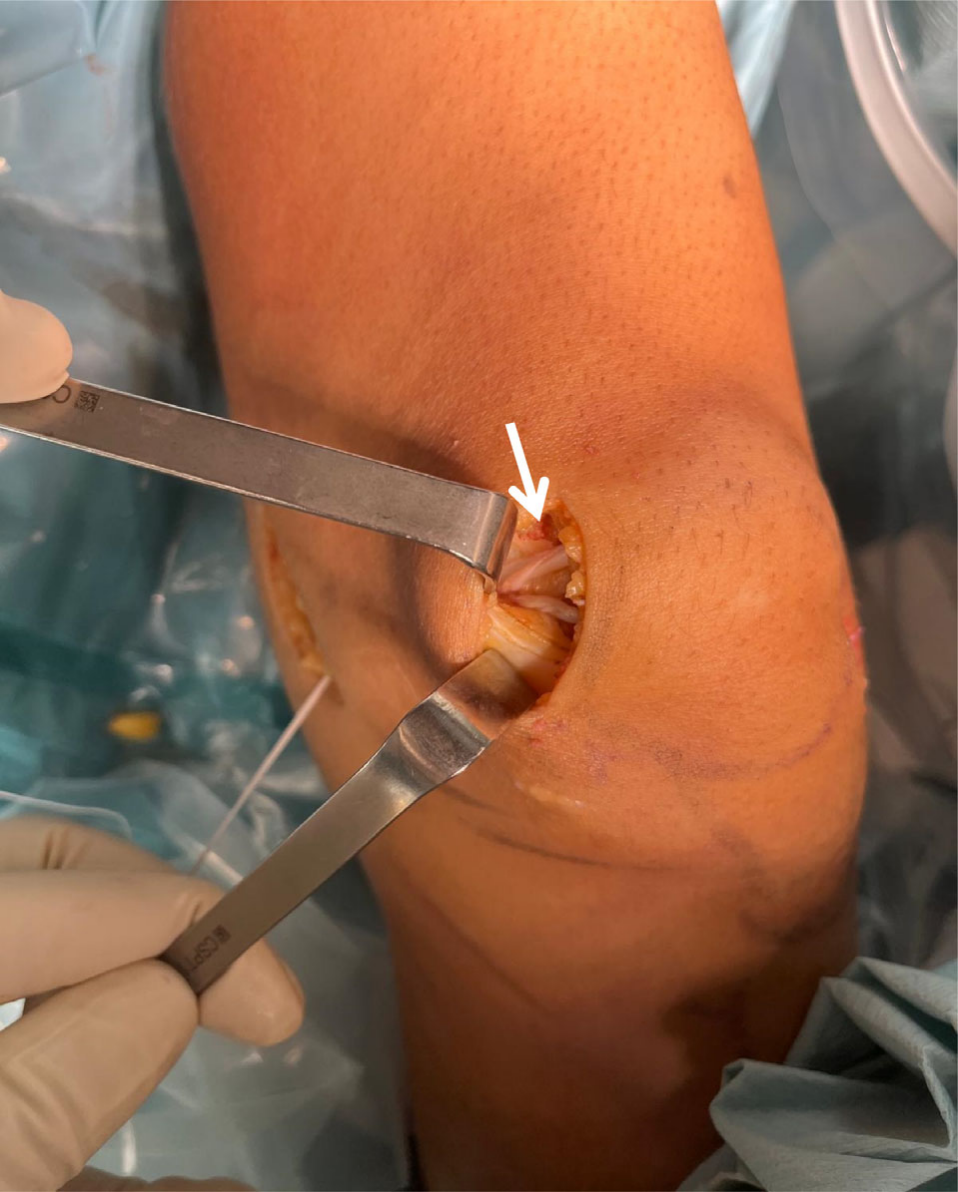
Final construct, anterior view of left knee. After passing the graft through the patellar tunnels, the free end is sutured to the exiting limb (white arrow) using high‐strength nonabsorbable sutures (FiberWire; Arthrex, Naples, FL).

With the knee positioned at approximately 30° of flexion to replicate functional loading conditions, graft tension is carefully adjusted to maintain the patella centered within the trochlear groove, allowing for approximately 10 mm of lateral translation under manual stress. The graft is then securely fixed using high‐strength, nonabsorbable sutures (FiberWire; Arthrex, Naples, FL) by anchoring both limbs—the entering strand at the proximal tunnel and the exiting strand at the distal tunnel. This technique avoids the need for implants or anchors (Figure 5).

### Final Assessment and Closure

Patellar tracking is evaluated throughout the full range of motion to confirm appropriate graft tension and alignment. The surgical wounds are thoroughly irrigated and closed in layers using absorbable sutures. No drain is placed (Table 2).

**TABLE 2:** Advantages and Limitations of the Technique

**Advantages**	**Limitations**
• Preservation of gracilis distal insertion maintains native vascularity and may enhance graft integration • Avoids need for femoral tunnel drilling and fluoroscopy • Minimally invasive: double incision, reduced soft tissue trauma • Implant‐free technique reduces cost and hardware‐related complications • Suitable for skeletally immature patients (no risk to femoral physis)	• Quasianatomic femoral fixation does not replicate the true anatomic femoral footprint • Patellar tunnels must be created, which carries a small risk of fracture • Gracilis tendon identification may be challenging in obese patients

### Postoperative Rehabilitation

Full range of motion and partial weight‐bearing with crutches are allowed immediately, as tolerated. Isometric quadriceps activation and early mobility exercises begin on day 1.

From week 6, closed‐chain strengthening is introduced. Full return to sport is typically allowed from the 4^th^ month, based on functional recovery.

## DISCUSSION

The overall efficacy of MPFL reconstruction in managing recurrent patellofemoral instability is well supported in the literature. Multiple studies have shown that MPFL reconstruction significantly reduces the risk of redislocation compared with nonoperative treatment or isolated repair techniques.^5^ Reported recurrence rates after reconstruction are generally low, ranging from 2% to 10%, depending on patient selection, surgical technique, and associated anatomical risk factors.^6^ Functional outcomes are also favorable with high patient satisfaction, restoration of patellar stability and return to preinjury activity levels in the majority of cases.^7^ However, failures have been associated with technical errors such as nonanatomic graft placement, particularly on the femoral side, or overtensioning of the graft.^8^ These findings underscore the importance of restoring proper patellofemoral biomechanics following MPFL reconstruction, whether through bone tunnel techniques or soft‐tissue‐based quasianatomic methods, to ensure consistent clinical outcomes.

This MPFL reconstruction technique introduces the concept of preserving the distal insertion of the GT, which may offer several theoretical and practical advantages over traditional methods. By maintaining the tendon's native tibial attachment, vascular supply is preserved, potentially enhancing graft viability and biological integration. In addition, this approach eliminates the need for a third distal incision, reducing soft tissue trauma, postoperative pain, and surgical time. The use of a single medial approach, combined with implant‐free fixation, also decreases operative cost and morbidity, making it a minimally invasive and reproducible option for the treatment of recurrent patellar instability.

This method builds upon the principles of quasianatomic reconstruction, which seeks to replicate the native path of the MPFL without the need for rigid bone tunnel placement, particularly at the femoral site.^9^ Previous studies have shown that using the adductor magnus tendon as a pulley to simulate the femoral attachment can achieve near‐isometric behavior of the graft, with good clinical outcomes and reduced complication rates.^10^, ^11^ By avoiding femoral drilling—especially in skeletally immature patients or in cases where intraoperative fluoroscopy is not available—this soft tissue‐based approach offers a reliable alternative that respects individual anatomical variability.^12^

As with any surgical technique, proper execution requires close attention to detail. A key technical pearl is the accurate identification of the GT, which can be more challenging in obese patients due to increased soft tissue depth, often requiring additional dissection. Although graft length has not been a limiting factor in our clinical experience to date, there is a theoretical risk that the tendon may be too short to complete the full trajectory in certain individuals and it may be necessary to release its tibial insertion, converting the technique to a standard free graft approach. There is no strict minimum graft length required, as the total length needed depends on individual patient morphology, size, and anatomical variability. However, based on our experience, we recommend that the harvested GT provide at least 16 cm of usable length to ensure it can be routed behind the adductor magnus and across the subretinacular plane to the patella without undue tension or the need for tibial detachment. Another important step is ensuring the graft passes smoothly behind the adductor magnus tendon without entrapment or twisting—this maneuver can be technically demanding. To simplify it, a Vicryl (Ethicon, Somerville, NJ) suture can be looped around the adductor magnus beforehand to guide graft passage. Finally, it is essential to maintain a minimum 10 mm cortical bridge between the two patellar tunnels to prevent the risk of iatrogenic fracture (Table 3).

**TABLE 3:** Pearls and Pitfalls

**Pearls**	**Pitfalls**
Preserve the gracilis tibial insertion to maintain vascularity and biological integrity	Misidentifying the gracilis tendon, especially in obese patients
Route the graft between layers 2 and 3 to protect the vastus medialis and facilitate native MPFL repair	Graft too short may require detachment of the tibial insertion
Create a ≥10 mm cortical bridge between patellar tunnels to reduce fracture risk	Narrow tunnel spacing increases risk of patellar fracture
Optional suture at adductor magnus may improve stability based on surgeon preference	Overtensioning the graft can lead to medial overconstraint and poor tracking

MPFL, medial patellofemoral ligament.

Potential limitations of this technique may be identified. First, it is a quasianatomic reconstruction, which means that although it aims to reproduce the functional path of the native MPFL, the femoral attachment is not anatomically replicated with a bony tunnel. Second, the technique requires the creation of patellar bone tunnels, which introduces a theoretical risk of fracture if not performed with adequate care and spacing. Lastly, identification of the GT can be technically demanding, particularly in patients with a high body mass index, where increased soft tissue depth may obscure landmarks and necessitate more extensive dissection.

This quasianatomic technique for MPFL reconstruction using an autologous GT with preservation of its tibial insertion offers a minimally invasive, implant‐free alternative to traditional approaches. By avoiding femoral tunnel drilling and maintaining native graft vascularity, the technique reduces surgical morbidity while respecting the functional anatomy of the MPFL. Although it presents certain technical challenges and does not provide a true anatomic femoral fixation, early clinical use suggests it is a safe, reproducible, and biologically favorable option—particularly suited for skeletally immature patients or those requiring soft tissue‐preserving strategies.

## DECLARATION OF GENERATIVE AI AND AI‐ASSISTED TECHNOLOGIES IN THE WRITING PROCESS

During the preparation of this work, the authors used ChatGPT (OpenAI) solely to assist with grammar correction and language refinement. No content generation or substantive contributions were made by the AI.

## DISCLOSURES

The authors (A.C., S.G‐G., G.G., P.M‐G., X.P.) declare that they have no known competing financial interests or personal relationships that could have appeared to influence the work reported in this article.
